# Global Analysis of *Chlorella variabilis* NC64A mRNA Profiles during the Early Phase of *Paramecium bursaria* Chlorella Virus-1 Infection

**DOI:** 10.1371/journal.pone.0090988

**Published:** 2014-03-07

**Authors:** Janet M. Rowe, Adrien Jeanniard, James R. Gurnon, Yuannan Xia, David D. Dunigan, James L. Van Etten, Guillaume Blanc

**Affiliations:** 1 Department of Plant Pathology, University of Nebraska, Lincoln, Nebraska, United States of America; 2 Nebraska Center for Virology, University of Nebraska, Lincoln, Nebraska, United States of America; 3 Laboratoire Information Structurale and Génomique UMR7256 CNRS, Aix-Marseille Université, Marseille, France; 4 Center for Biotechnology, University of Nebraska, Lincoln, Nebraska, United States of America; The Ohio State University, United States of America

## Abstract

The PBCV-1/*Chlorella variabilis* NC64A system is a model for studies on interactions between viruses and algae. Here we present the first global analyses of algal host transcripts during the early stages of infection, prior to virus replication. During the course of the experiment stretching over 1 hour, about a third of the host genes displayed significant changes in normalized mRNA abundance that either increased or decreased compared to uninfected levels. The population of genes with significant transcriptional changes gradually increased until stabilizing at 40 minutes post infection. Functional categories including cytoplasmic ribosomal proteins, jasmonic acid biosynthesis and anaphase promoting complex/cyclosomes had a significant excess in upregulated genes, whereas spliceosomal snRNP complexes and the shikimate pathway had significantly more down-regulated genes, suggesting that these pathways were activated or shut-down in response to the virus infection. Lastly, we examined the expression of *C. varibilis* RNA polymerase subunits, as PBCV-1 transcription depends on host RNA polymerases. Two subunits were up-regulated, RPB10 and RPC34, suggesting that they may function to support virus transcription. These results highlight genes and pathways, as well as overall trends, for further refinement of our understanding of the changes that take place during the early stages of viral infection.

## Introduction

Algae as a source for biofuels and other bioproducts are becoming increasingly important because some algae can produce fuel compatible lipids at a rate significantly higher than crop plants. Also certain algae can be grown in water and landscapes unsuitable for crop plants (e.g., [1]). However, it is predicted that pathogens, including viruses, will become an issue when algae are grown on a large scale that will tend to limit production yields. The field of algal virology is relatively new and only a few algal viruses have been characterized [2]–[7]. However, viruses infecting algae with ssRNA, dsRNA, ssDNA and dsDNA genomes are known [4]. Most algal virus research has focused on large dsDNA viruses that infect both freshwater and marine algae and are classified in the family *Phycodnaviridae*. The large (190 nm in diameter) icosahedral, dsDNA, plaque-forming *Paramecium bursaria* chlorella virus (PBCV-1) is the most studied phycodnavirus and it serves as the type strain for the genus *Chlorovirus* [6]. PBCV-1 contains an internal membrane that is surrounded by a glycoprotein containing capsid [8], [9]. The 331 kb PBCV-1 is predicted to have 416 protein-encoding genes and 11 tRNA-encoding genes [6].

PBCV-1 infects the small, coccoid, nonmotile, unicellular, eukaryotic, photosynthetic green alga, *Chlorella variabilis* NC64A, which is a host for many chloroviruses that are found in freshwater throughout the world. The 46-Mb *C. variabilis* nuclear genome is predicted to contain 9,791 protein-encoding genes [10]. The majority of the NC64A genes are related to terrestrial plant homologs, but it also encodes proteins that are only found in algae as well as a few genes of likely viral origin. Whereas most *Chlorella* species are naturally free-living, *C. variabilis* is a hereditary photosynthetic endosymbiont of the unicellular protozoan *Paramecium bursaria* [11]. This symbiosis is facultative because both the paramecium and *C. variabilis* can be grown separately in laboratory conditions.

Currently, the PBCV-1/*C. variabilis* system is one of the best models for studying virus infection in algae and much is known about the infection process [12]. However, a facet that remains essential to advancing our knowledge and managing virus infections is the means by which algal hosts defend and/or avoid virus infections. That such mechanisms exist is implied from the following observations: i) algae are resistant to most viruses, ii) some virus genes occur in host genomes, iii) host-initiated programmed cell death occurs in response to some virus infections [13], and genes involved with RNA silencing and their transcripts are present in certain algae [14], [15].

If an algal cell is going to mount a successful defense against a virus, it must do so quickly upon infection. In the case of PBCV-1, the virus attaches to and degrades the host cell wall at the point of entry [6], [12]. The viral DNA and virion-associated proteins enter the cell after the presumed fusion of the viral and host membranes. Once inside the host cell, circumstantial evidence suggests that the viral DNA quickly moves to the nucleus and commandeers the host transcription machinery; viral transcripts can be detected as early as 7 min post infection (p.i.) [16].

Homologs to *Arabidopsis* genes involved in RNA silencing and genes known to be induced by virus infection are present in the *C. variabilis* genome [15]. Transcripts for a majority of these homologs appear before and during the first hour of PBCV-1 infection. RNA directed DNA methylation and Sense post-transcriptional gene silencing were identified as possibly being used by *C. variabilis* for virus defense and/or gene regulation, highlighting the similarities between green algae and vascular plants.

Because the early stages of infection are critical for determining the ultimate fates of both the host and virus (including any virus progeny), an RNA-Seq study of *C. variabilis* cells infected by PBCV-1 was conducted to understand the global dynamics of *C. variabilis* gene expression during infection. cDNAs from poly(A+)-containing RNAs isolated from cells at six time points during the first hr of infection were sequenced using an Illumina sequencing platform (0, 7, 14, 20, 40 and 60 min p.i. [referred to as T0, T7, T14, T20, T40 and T60, respectively)]. A total of 105 million single-end 50-bp reads were generated and mapped to both the host and virus genomes; this data was used to estimate the expression of all genes. Two important issues regarding host transcription are addressed: i) What is the timing of changes in the reprogramming of the host transcriptional machinery? ii) Which genes and functional pathways respond to virus infection? A global analysis of PBCV-1′s transcripts using the same datasets is presented in a companion manuscript [16] that addresses specific questions regarding virus transcription. To our knowledge these are the first global analyses of an algal host and a virus during infection in a laboratory setting.

## Results and Discussion

### Divergent Dynamics of Cohabiting Transcriptomes

**Figure 1:**
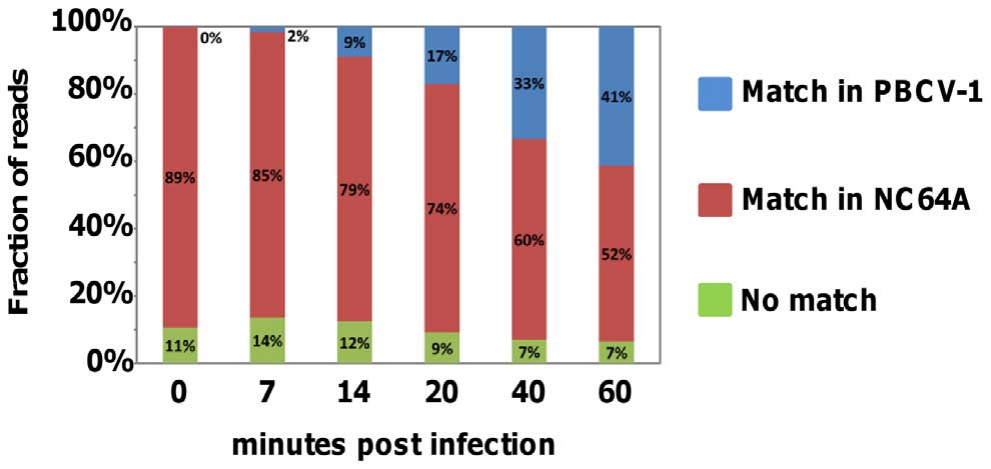
Frequency distribution of mRNA reads mapped to the virus PBCV-1 and host *C. variabilis* genomes. Reads listed as no match probably correspond to transcript sequences that overlap with exon junctions, possible contaminations and/or reads containing sequencing error.

The mRNA profiles measured by Illumina sequencing of control and virus infected cells indicate that the majority of sequence reads mapped to either the *C. variabilis* or PBCV-1 genomes in the T0 to T60 datasets (Table S1). A small percentage of sequence reads (7 to 14%) did not produce significant matches with the reference genomes and were labeled as “no match” (Fig. 1 and [16]). These reads probably correspond to transcript sequences that overlap with exon junctions (i.e., DNA sequences separated by an intron in the genome) that were not identified by TOPHAT. There was a rapid decrease in the proportion of sequenced host transcripts, mirrored by an increase in the proportion of viral mRNAs during the first hr of infection. By 60 min p.i. the percentages of identifiable mRNAs were relatively close between the host and the virus (i.e., 52% versus 41%, respectively). The divergent dynamics of changes in the host and virus mRNAs indicate a rapid reprogramming of the host transcription machinery to transcribe viral DNA. Details on how reprogramming occurs are unknown but at least two factors are involved. i) Host chromosomal DNA is degraded into 150–200 kb pieces within minutes after infection, presumably by PBCV-1 encoded and packaged DNA restriction endonucleases [17]. ii) PBCV-1 encodes a SET domain-containing protein (referred to as vSET) that methylates Lys-27 in histone 3 [18]. vSET is packaged in the PBCV-1 virion [19], and circumstantial evidence indicates vSET represses host transcription following virus infection [20]. Consequently, PBCV-1 has substantial means, including chromatin degradation and chromatin remodeling, to interfere with host gene expression and rapidly commandeer the host transcription machinery to transcribe virus genes. The proportional decrease in host transcripts over time is probably the result of several processes: i) an increase in transcription of the viral genome; ii) a possible higher rate of degradation of host transcripts; and iii) a decrease in the number of host transcripts due to a slowdown of host gene transcription. Indeed a previous study reported that the amount of total RNA synthesized in the cell after PBCV-1 infection decreases progressively relative to non-infected cells [21].

### Normalization and Interpretation of the Data

The opposite trajectories of host and virus mRNA abundances during infection as well as the complex transcriptional regulation events are significant issues in the choice of an appropriate method for normalizing read counts. Most normalization methods that are developed to correct for sequencing depth rely on the key assumption that most genes are not differentially expressed between collection points [22]. Obviously, this condition is not met in the case of PBCV-1 infected *C. variabilis* cells or in many host-virus interactions. Changes taking place in host transcription during infection result from several effects: i) a global decrease in transcription activity of cellular genes induced by viral factors with systemic action (i.e., endonucleases and histone methylation); ii) changes in the expression of specific host genes induced by viral factors with targeted action (e.g., PBCV-1 encodes at least 4 putative transcription factors); and iii) changes in the expression of host specific genes in response to viral infection. Thus, it is likely that the cellular concentration of all host transcripts decreases during infection; however, the dynamics of the decrease may vary from one gene to another, depending on the targeted regulatory events initiated by the virus or the host. This represents a general problem in host-pathogen interactions, and especially in virology.

No normalization method exists that can correct read counts by taking into account the global decrease of transcriptional activity using our data. Therefore, we applied a method implemented in the DESeq package that essentially corrects read counts for sequencing depth [23]. This method balances read counts between datasets such that the overall transcriptome sizes at each time point are artificially constant. With this method, the global transcriptome decrease in the host is masked, and only expression changes that alter the relative abundance of mRNAs across time points are measured. It is important to point out that this transcriptome analysis estimates mRNA abundance and that transcriptional expression changes cannot be inferred directly. It is also important to note that assuming the size of the mRNA pool does not change during the course of infection may not reflect reality for the reasons listed above.

### Host Transcription

**Figure 2:**
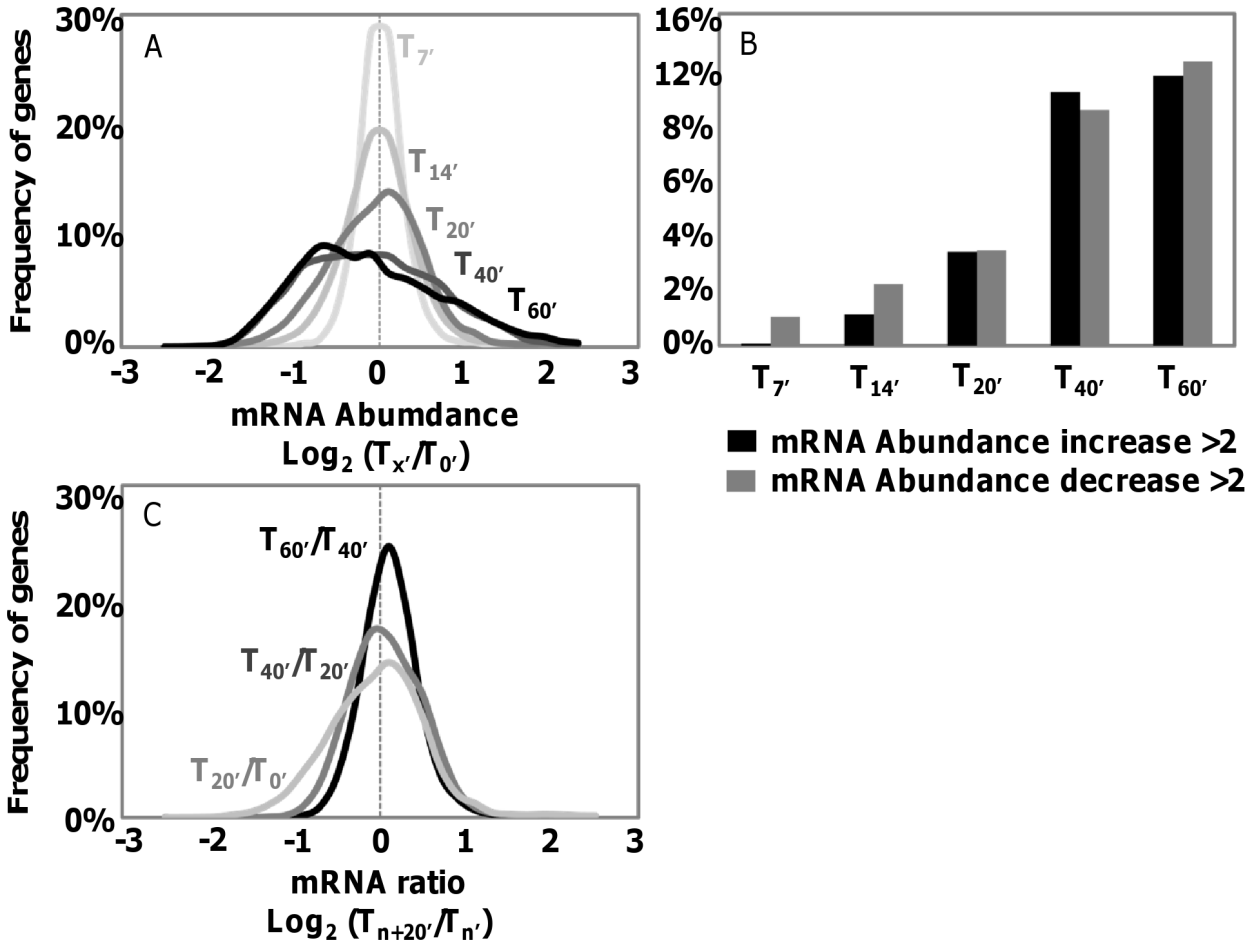
Global *C. variabilis* mRNA changes during virus PBCV-1 infection. (A) Frequency distributions of log_2_ abundance ratios for genes between datasets T0 and T7–T60. (B) Frequency of genes with absolute mRNA changes relative to T0>2 fold. (C) Frequency distributions of log_2_ abundance ratios for genes between datasets T_n_ and T_n+20′_.

After read count normalization, we analyzed changes in the abundance of mRNAs relative to T0. The magnitude of expression changes increased over time (Fig. 2A). This trend was reflected by a flattening of the mRNA abundances level ratio curves with increasing times from T0. The proportions of host genes with absolute fold changes ≥2 at T7, T14, T20, T40 and T60 were 1.5%, 4.5%, 9.1%, 23.6% and 26.7% respectively, with an approximately equal distribution between up- and down-regulated genes (Fig. 2B). The most significant mRNA changes occurred within the first 20 min p.i. (i.e., widest distribution for T20/T0 in Fig. 2C). In contrast, the T60/T40 log abundance ratio distribution formed a sharper peak around zero, indicating that the mRNA amounts did not change markedly between 40 and 60 min p.i. This relative transcriptome stasis was already apparent in Fig. 2A where the distribution curves for T40 and T60 are superimposed. The levels of mRNA abundance between 20 and 40 min (T20/T40 curve in Fig. 2C) exhibited an intermediate range of ratios.

The transcriptome analysis presented here revealed two important features about host mRNA profiles. First the beginning of the transcriptional response takes place within a few minutes after the virus enters the cell. A new genetic program was established within the first 40 min p.i. and appears to remain relatively constant until at least 60 min p.i. Second, the number of genes whose expression changed during infection was substantial – i.e., the abundance of transcripts of 2,149 genes (26.7%) changed by at least 2 fold between T0 and T60. Thus infection by PBCV-1 triggers a significant remodeling of the host transcriptional landscape.

### mRNA Profiling

**Figure 3:**
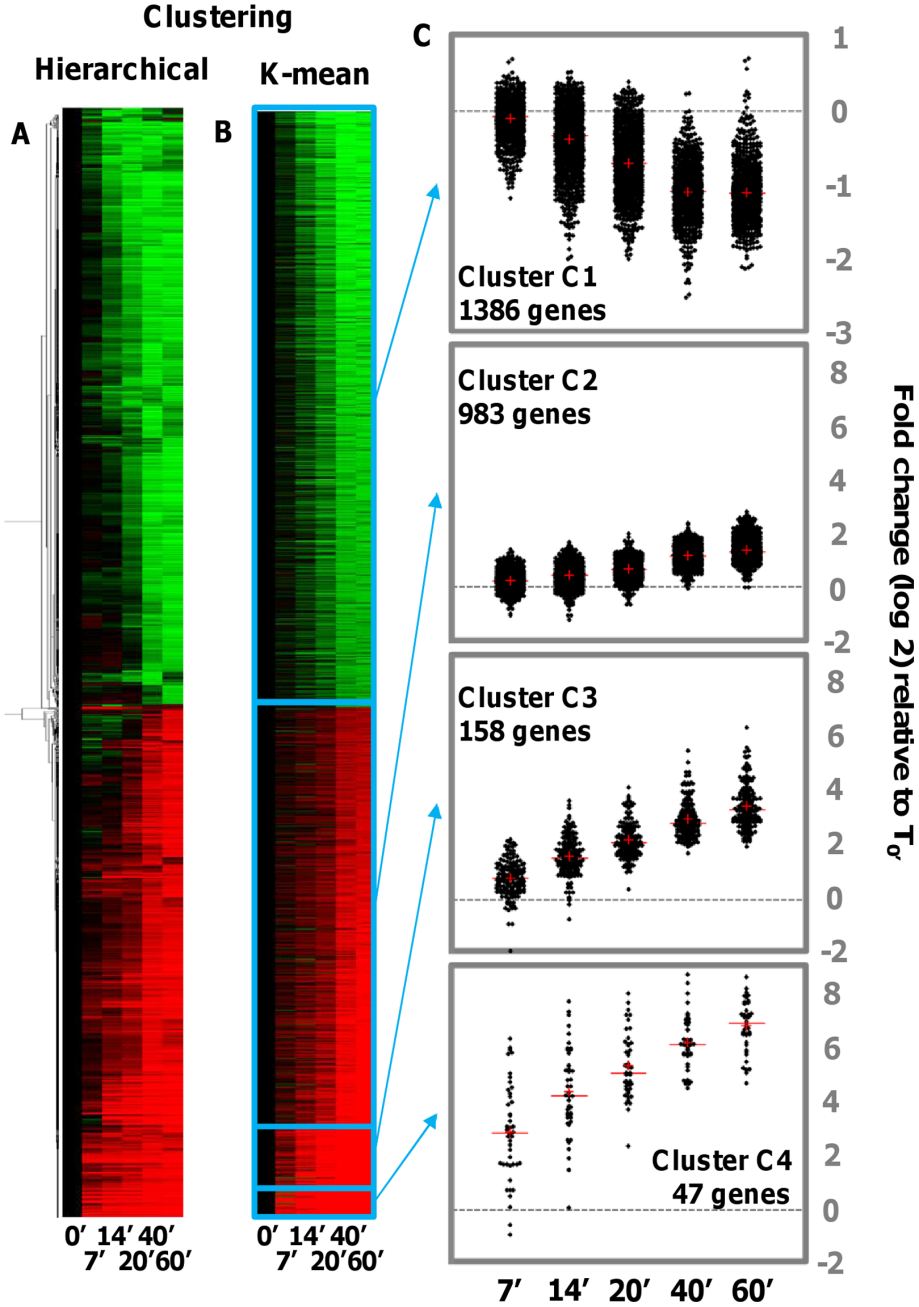
*C. variabilis* mRNA profiling of host genes during infection. (A) Heat map of normalized mRNA abundance levels for 2,574 host genes with absolute fold change >2 relative to T0 in at least one time point (T7 to T60) and hierarchical clustering tree (left) based on the uncentered correlation distance. (B) Heat map of normalized abundance levels sorted according to 4 clusters defined using K-means clustering. (C) Scattergrams of log_2_ abundance level ratios relative to T0 for each K-means cluster and dataset.

Of *C. variabilis*’s 9,792 predicted protein-encoding genes, 16 did not have any read attributed to them at any time during infection. An additional 1,867 genes had less than 50 reads at all time points and were discarded from further analyses after being designated as too weakly expressed. After normalization, transcripts from 5,335 genes had <2-fold changes relative to T0 across all time points of infection. The transcript profiles of the remaining 2,574 genes exhibited ≥2-fold changes relative to T0 for at least one time point (T7 to T60). They were analyzed using clustering methods. Hierarchical clustering readily separated gene products that were globally up- or down-regulated during the first hour of infection (Fig. 3A). However, no obvious sub-clusters containing more specific patterns of mRNA abundances were apparent from the heat map and clustering tree. The same data set was more objectively segmented using the k-means clustering method to investigate if more complex mRNA abundance patterns were shared between genes. As shown in Fig. 3B, the 2,574 genes grouped into four k-mean clusters: cluster C1 contained all genes that exhibit decreasing mRNA levels while clusters C2, C3 and C4 contained genes with globally increasing mRNA levels (Fig. 3C). Genes in clusters C2, C3 and C4 varied more by the intensity of their increases in mRNA abundance than by the overall shape of their expression profiles. Thus analyses of the changes in the transcripts of the 2,574 most variable host genes only identified two simple patterns: a progressive increase in mRNA abundance or a decrease in mRNA abundance during the first hour of infection. Table S2 presents a list of genes with transcript abundances that change during virus infection and to what degree they each change.

### Evidence for Pathway Regulation

Based on the major trends in mRNA abundances outlined above, the 2,574 most variable host genes were divided into two groups: down-regulated genes (C1) and up-regulated genes (C2+C3+C4). NC64A proteins were also sorted into functional categories using the KEGG module classification system [24], [25]. For each regulation group and metabolic pathway, the significance of the difference between the observed number of up- or down-regulated genes versus the number expected by chance was statistically tested (Table 1). Three functional modules have significantly more up-regulated genes than expected by chance (p≤0.01): cytoplasmic ribosomal proteins (M00177; p ∼0), jasmonic acid biosynthesis (M00113; p* = *3.3E-5) and anaphase promoting complex/cyclosome (APC/C; M00389; p* = *0.0039). Down-regulated genes were overrepresented in the shikimate pathway (M00022; p* = *0.0014) and some of the components of the spliceosome small nuclear ribonucleoproteins (snRNP; a collection of 4 overlapping KEGG functional categories: M00351, M00352, M00354 and M00355; *P* = 0.0016). Individual mRNA profiles of genes for each of these five functional modules are shown in Fig. S1A–E. The identification of potentially regulated pathways allowed us to make assumptions about the mechanisms and strategies used by both partners. PBCV-1 is known to encode at least four transcription factors that can potentially be involved in the control of host genes [26].

**Table 1:** KEGG functional module with overrepresented up- or down-regulated genes.

KEGG Module ID	Functional Category	Number of Differentially Regulated Genes	Number of Genes in Category	p-value^a^
Up-regulated group				
M00177	Ribosome, eukaryotes	56	73	0
M00113	Jasmonic acid biosynthesis	7	10	3.3E-05
M00389	APC/C complex	5	10	0.0039
Down-regulated group				
M00022	Shikimate pathway	5	6	0.0014
M00351+M00352+ M00354+M00355	Spliceosome snRNP complex	23	64	0.0016

a Binomial test. Note that because genes often belong to more than one KEGG category, a correction for multiple testing such as the Bonferroni correction could not be applied.

Cytoplasmic ribosomal proteins had the highest number of up-regulated genes (56 genes out of 73 identified genes). Of the 17 remaining genes, 14 also have increased mRNA profiles but the maximal increase relative to T0 did not exceed 2 fold (Fig. S1A). The up-regulation of host cytoplasmic ribosomal protein genes could be a response driven by the virus to maintain a sufficiently large pool of ribosomes for synthesis of the viral proteins. Also, the down-regulation of genes involved in spliceosome functions (Fig. S1E) may favor expression of virus genes over those of the host, since most of the host genes contain introns [10]. Our RNA-seq study of PBCV-1 mRNA abundances revealed that several virus transcripts were probably erroneously excised, most likely as a result of the activity of host spliceosomes [16]. Reducing the splicesome pool could be an adaptive strategy of the virus to limit viral transcript excision. APC/C is a multi-subunit ubiquitin ligase complex with essential roles in cell cycle regulation. Some DNA viruses manipulate APC/C to promote an S phase-like state in the host cell that is likely to support viral genome replication [27]. The observation that NC64A APC/C genes were up-regulated upon infection (Fig. S1C) suggests that PBCV-1 may also manipulate the APC/C complex to support its replication.

Jasmonates (a collective term for jasmonic acid and its derivatives) are lipid-derived molecules in plants that are activated by both abiotic and biotic stressors (including microbial pathogens), and also play roles in developmental processes [28]. The *C. variabilis* genome encodes at least 12 proteins homologous to plant enzymes involved in jasmonic acid biosynthesis [10]. Seven of these were considered up-regulated (Fig. S1B). Thus far, minimal research has been done in regards to algae and jasmonate signaling. Mostly, the research has focused on the response of multicellular species to jasmonic acid. Within the Chlorophyta both *Chlorella vulgaris* and *Haematococcus pluvialis* respond to jasmonic acid and *Scenedesmus incrassulatus* responds to methyl ester jasmonic acid [29]–[31]. However, these studies do not reveal anything about green algae having a jasmonate signaling pathway, only that they exhibit growth and metabolic (and to a limited extent pathogen defense and transcription) responses to these compounds. Though the up-regulated expression of these 7 genes is not a definitive link to jasmonate signaling in *C. variabilis*, it may indicate a more primitive form of this process in green algae, which is consistent with the limited jasmonate response studies.

Down-regulation of the genes involved in the shikimate pathway (Fig. S1D) is more difficult to interpret in the context of viral infection. This pathway links metabolism of carbohydrates to the biosynthesis of aromatic compounds, which are precursors of aromatic amino acids and aromatic secondary metabolites. The likely effect of a down-regulation of the shikimate pathway is to reduce the pool of aromatic amino acids necessary for the synthesis of viral proteins. Thus the shutdown of the shikimate pathway could be a systemic host-driven defense mechanism to interfere with secondary metabolites involved in host responses to virus replication and propagation.

### Host Virion Protein

In addition to pathways with excess in up or down regulated genes, we examined the abundance of host transcripts that might play a role in virus replication. Among them is a host-encoded protein that is packaged in the PBCV-1 virion [19]. This 101 amino acid residue protein (EFN53917) resembles members in the high mobility group (HMG) box superfamily of DNA binding proteins. HMG box-containing proteins are involved in regulation of DNA-dependent processes, such as transcription, replication, and DNA repair, all of which require changing chromatin conformation. Thus, this virion-associated host protein may be important in initiating PBCV-1 gene expression. Using our RNA-seq dataset, we found that its mRNA level was moderate in uninfected cells; thus, no evidence for its specific up-regulation occurred during the first hour of infection (Fig. S1F). It cannot be ruled out that the gene is up-regulated at a later stage of infection; however this seems unlikely as the host genome continues to be degraded by endonucleases during infection, diminishing the chances of retaining a functional gene. Instead, our results suggest that the virus relies on the existing pool of mRNA at the early stage of infection to incorporate the encoded protein into the virion. Another possible scenario is that the protein expression is regulated at a post-transcriptional stage.

### RNA Polymerases

Like all eukaryotes, *C. variabilis* possesses 3 distinct, multi-subunit RNA polymerases (RNAPs). RNAP I synthesizes the rRNA precursor, RNAP II transcribes mRNAs and small non-coding RNAs, and RNAP III produces tRNAs and other small RNAs. Unlike its eukaryotic host, PBCV-1 does not encode a functional RNAP. Viruses in the family *Phycodnaviridae*, together with those in the *Poxviridae*, *Iridoviridae*, *Ascoviridae*, *Asfarviridae* and *Mimiviridae* familes are believed to have a common evolutionary ancestor and are referred to as nucleocytoplasmic large DNA viruses (NCLDV) [32]–[34]. All the sequenced NCLDVs, with the exception of the Chloroviruses, Prasinoviruses and Phaeoviruses (3 genera in the family *Phycodnaviridae*), encode up to 6 RNAP subunits [35]. Phylogenetic evidence suggests that the phycodnavirus ancestor encoded the RPB1 and RPB2 subunits of RNA Pol II [32], which form the opposite sides of the active center cleft, and that the genes are subsequently absent in the lineage leading to the Chloroviruses, Prasinoviruses and Phaeoviruses, suggesting gene loss. These gene losses must have had a significant effect on the virus lifestyle as their descendants then had to rely on the host transcription machinery. The details on how chloroviruses interact and control the host RNAP complexes are unknown. This prompted us to investigate the expression of the host RNAP subunit genes.

Of the 25 RNAP subunit genes identified in *C. variabilis* (Table S3 and Fig. S1G), only two were in the up-regulated K-means clusters. They included a gene encoding RPB10, a subunit that plays an essential role in the assembly and maintenance of integrity of RNAPs I–III [36], and a gene encoding RPC34, another essential subunit involved in the recruitment of RNAP III to the pre-initiation complex [37]. Interestingly, the RPB10 gene also exists in some NCLDV members including *Emiliania huxleyi* virus, *Cafeteria roenbergensis* virus, *Phaeocystis globosa* virus and an organic lake phycodnavirus. Thus the apparent up-regulation of the RPB10 gene during PBCV-1 infection suggests a virus-driven adaptive mechanism to relieve the loss of the corresponding gene in the PBCV-1 ancestor. In contrast, the host RPB1 and RPB2 mRNA populations did not change during infection.

Seven genes encoding subunit proteins associated with all three RNAP types belonged to the down-regulated K-means cluster. Except for RPB6 that is involved in core polymerase complexes, the down-regulated RNAP subunits are involved in accessory functions, including initiation complex formation and stabilization, start site selection and transcription termination. These proteins also include 3 of the 4 homologs to RNAP subunits that are dispensable for yeast survival [38], suggesting that they have no critical role in core RNA synthesis. However their down-regulation may alter initiation and termination processes. This may explain why read-through transcripts for some chlorovirus genes are observed at latter stages of PBCV-1 infection [39].

## Conclusions

This is the first examination of an algal host’s global transcriptional response to virus infection in a laboratory setting. Unpredictable changes in the global mRNA population during infection pose a problem for normalizing data and thus a problem for proper assessment of what perceived changes in mRNA abundance mean. Even with restrictions on interpretation, our global analysis reveals two important changes that occur in mRNA populations during the early stage of infection. Namely, a rapid transcriptional response of host genes upon virus infection: significant changes in the global host transcriptome occurred as soon as 7 min p.i. Also, groups of genes exhibit different dynamic changes in mRNA abundance suggesting that they respond differently to infection. This differentiation in responses implies that genes are under the control of different regulators trying to activate or deactivate certain metabolic pathways. This global response is even more difficult to understand because it results from the interplay between two competing and antagonist mechanisms: a defensive reaction from the host and a takeover of the host metabolism by the virus. This information can be used to further refine future analytical and statistical experiments so that both new global and targeted approaches can result in more robust interpretations of infection data.

Through statistical assessment of changes in mRNA abundance and functional gene categories, we identified several significantly regulated functional categories, some of which are consistent with what we would expect to occur during virus replication (e.g., mRNAs for ribosomal proteins). mRNA abundance increases also occurred for genes involved in jasmonic acid and the APC/C complexes, as well as decreases for shikimate pathway and parts of the RNA polymerase complexes. No increase in mRNA abundance occurred for the main RNAP subunits (RPB1 and RPB2) that are encoded in the majority of NCLDV members except for some phycodnaviruses, including PBCV-1. PBCV-1 appears to rely on the existing pool of RNAPs for its own transcription. However this pool is probably sufficient given that many host genes presumably stop being transcribed, which would reduce competition between the host and virus genomes to gain access to the available RNAPs.

There is some similarity in the transcriptional response of the *C. variabilis*/PBCV-1 system with the response of *Emiliania huxleyi* during virus EhV infection. Pagarete, et al. [40] used a microarray analysis to examine expression of ∼3,500 *E. huxleyi* genes and infecting viruses (also members of *Phycodnaviridae*) during a mesocosm algal bloom experiment. Of the 81 up-regulated genes corresponding to host transcripts, the majority was from genes with unknown function, and the remainder belonged to 14 different KEGG functional classes. In this study the majority of the annotated up-regulated genes fell into 3 functional classes: 1) translation, ribosomal structure and biogenesis 2) energy production and conversion and 3) lipid transport and metabolism.

Lastly, it is important to compare these findings with those of a targeted analysis of RNA silencing and potential defense genes using the same dataset. Previously, it was reported that *C. variabilis* encodes 375 homologs to genes involved in RNA silencing (beyond core machinery) and to genes induced during viral infection in higher plants. The majority of these genes are expressed in healthy and infected (up to 60 min. p. i.) host cells with over a quarter of them experiencing ≥2-fold changes [15]. Though the collection of homologs to genes involved in higher plant responses to infection do not present an obvious pathway or mechanism, combined with the significantly affected functional categories detected herein, future studies can begin piecing together how the identified genes play into the overall scheme of virus takeover and host response. Furthermore, it is important to note that both targeted and global analyses are vital for understanding virus-host interactions. Our global analyses herein did not highlight those genes involved in RNA silencing, yet our previous, targeted assessment demonstrated their likely importance in host functioning and/or virus response. RNA directed DNA methylation and Sense post-transcriptional gene silencing were identified as strong candidate pathways [15]. Though databases provide an excellent opportunity for managing large amounts of data, which cannot be assessed in total by an individual, there is a limit to what these collections can tell us with regard to any specific system and individual assessment cannot be replaced. Therefore, a complete picture of virus-host interactions will require a combined approach.

## Materials and Methods

### Culture Conditions and Sample Preparation

The culturing of *C. variabilis* NC64A and PBCV-1, and virus purification have previously been described [41]. Sample collection, preparation and Illumina sequencing were also described elsewhere [15], [16].

### Transcriptome Analysis

Raw read sequences were deposited at NCBI’s BioProject database under accession SRP026413. Read alignment, statistics, and clustering analyses were described previously [15]. Briefly, BOWTIE2 [42] was used to align reads onto the PBCV-1 (Genbank: NC_000852) and NC64A (Genbank: ADIC00000000) genomes, simultaneously, while TOPHAT2 [43] was used to align reads that spanned exon junctions (Table S2 and see also [16]). CLUSTER and TREE-VIEW [44] were used for clustering analysis and visualization of results. The DESeq method was used to normalize read counts to library size at T = 0 [23]. Assignment of NC64A proteins to KEGG pathways was performed through the KEGG KAAS web tool [24]. Genes targeted for analysis based on expression level changes were further annotated based on current *C. variabilis* genome annotation and closest significant homology (e-value ≤1e^−5^) in the NCBI and Swiss-Prot databases.

## Supporting Information

Figure S1 **NC64A mRNA profiles for selected genes.** Genes that have mRNA level ratios >2 fold and <2 fold in at least one time point relative to T0 are represented by red and green lines respectively. Genes with lower mRNA level changes are indicated by a black line.
(PDF)

Table S1 **Genomic location of mapped reads.**
(DOCX)

Table S2 **Read counts for** ***C. variabilis*** **genes.**
(XLSX)

Table S3 **RNAP subunits transcription.**
(DOCX)
