# Supplementary material for: Global Analysis of Chlorella variabilis NC64A mRNA Profiles during the Early Phase of Paramecium bursaria Chlorella Virus-1 Infection
Source: PLoS One. 2014 Mar 7;9(3):e90988. doi: 10.1371/journal.pone.0090988 (PMC3946773; doi:10.1371/journal.pone.0090988)
Supplement: Figure S1 — NC64A mRNA profiles for selected genes. Genes that have mRNA level ratios >2 fold and <2 fold in at least one time point relative to T0 are represented by red and green lines respectively. Genes with lower mRNA level changes are indicated by a black line. (PDF) [file pone.0090988.s001.pdf]

Figure S1

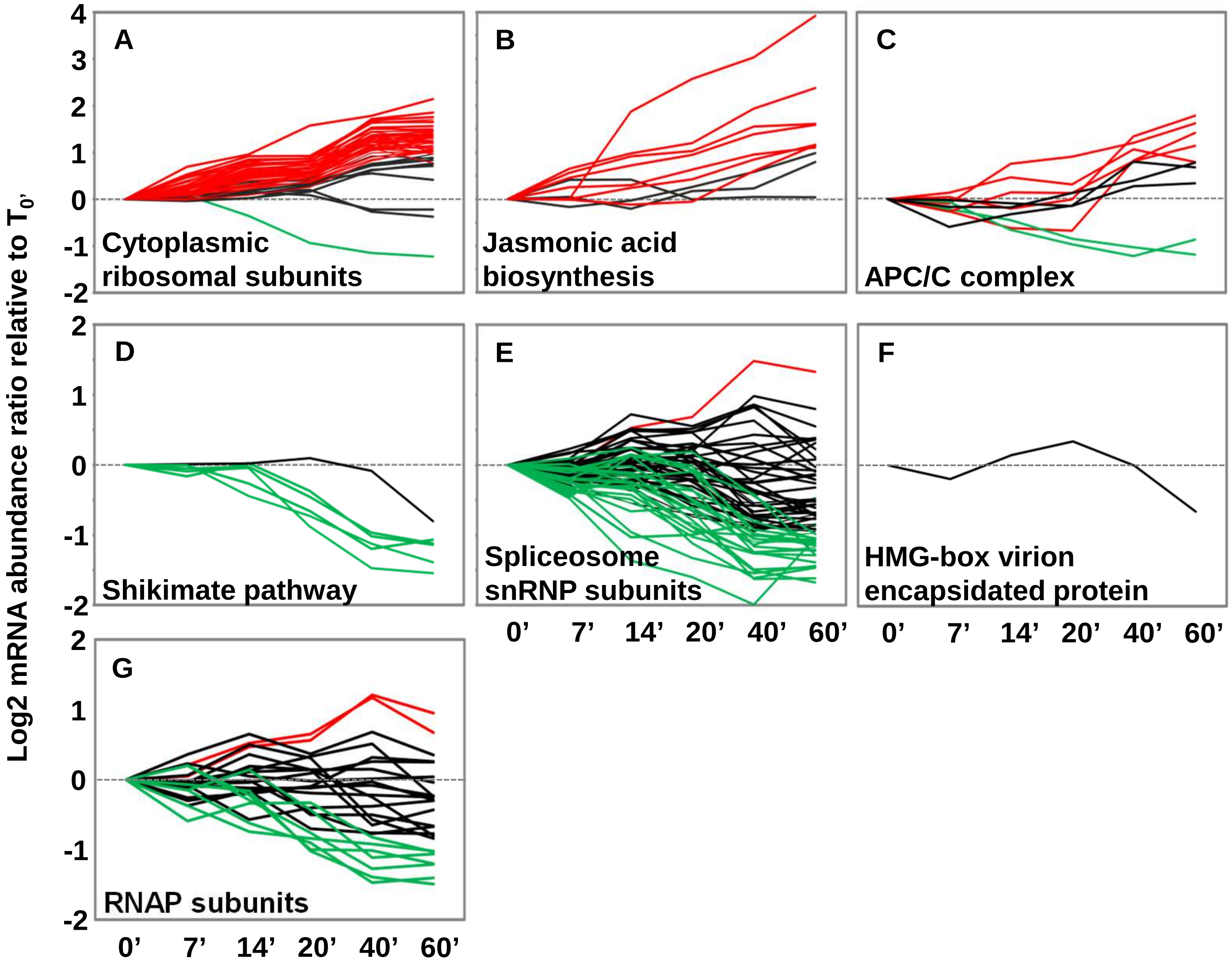

Figure S1: NC64A mRNA profiles for selected genes.
Genes that have mRNA level ratios >2 fold and <2 fold in at least one time point relative to T0 are represented by red and green lines respectively. Genes with lower mRNA level changes are indicated by a black line.
