# Supplementary material for: Global Analysis of Chlorella variabilis NC64A mRNA Profiles during the Early Phase of Paramecium bursaria Chlorella Virus-1 Infection
Source: PLoS One. 2014 Mar 7;9(3):e90988. doi: 10.1371/journal.pone.0090988 (PMC3946773; doi:10.1371/journal.pone.0090988)
Supplement: Table S1 — Genomic location of mapped reads. (DOCX) [file pone.0090988.s002.docx]

Table S1: Genomic location of mapped reads

| Time post infection | PBCV-1 | | |  | *C. variabilis* NC64A | | | Unmapped reads | Total |
| --- | --- | --- | --- | --- | --- | --- | --- | --- | --- |
|  | genome | exon junctions | repeated sequences‡ |  | genome | exon junctions | repeated sequences‡ |  |  |
| 0' | 44 | 0 | 4 |  | 10,722,808 | 1,695,135 | 1,712,784 | 1,701,558 | 15,832,333 |
| 7' | 254,039 | 32 | 18,253 |  | 11,277,126 | 1,725,480 | 1,819,808 | 2,385,251 | 17,479,989 |
| 14' | 1,506,952 | 412 | 81,077 |  | 10,580,234 | 1,639,300 | 1,705,375 | 2,205,123 | 17,718,473 |
| 20' | 2,836,982 | 1218 | 144,079 |  | 9,772,768 | 1,496,441 | 1,692,515 | 1,615,691 | 17,559,694 |
| 40' | 5,413,608 | 2836 | 306,260 |  | 7,536,772 | 1,118,816 | 1,545,862 | 1,190,574 | 17,114,728 |
| 60' | 7,741,030 | 2530 | 376,628 |  | 7,520,250 | 1,086,744 | 1,626,652 | 1,298,363 | 19,652,197 |

‡Reads that produced more than one alignment with the reference sequence.
