# Supplementary material for: Global Analysis of Chlorella variabilis NC64A mRNA Profiles during the Early Phase of Paramecium bursaria Chlorella Virus-1 Infection
Source: PLoS One. 2014 Mar 7;9(3):e90988. doi: 10.1371/journal.pone.0090988 (PMC3946773; doi:10.1371/journal.pone.0090988)
Supplement: Table S3 — RNAP subunits transcription. (DOCX) [file pone.0090988.s004.docx]

Table S3. RNAP subunits transcription

| **Genbank accession number** | **RNAP complex** | **Subunit name**  **(including synonyms)** | **Function** | **Gene dispensability**  **in yeast**‡ |
| --- | --- | --- | --- | --- |
| *Upregulated genes* | |  |  |  |
| EFN58690 | I+II+III | RPB10, POLR2L, RPABC5 | Polymerase Core | essential |
| EFN57447 | III | RPC34, POLR3F, RPC6 | Open complex stabilization | essential |
| *No differencial expression* | | | | |
| EFN59491 | I | RPA2, POLR1B | Active center | essential |
| EFN56415 | I | RPA43 | Initiation complex formation | essential |
| EFN55686 | I | RPA1, POLR1A | Active center | essential |
| EFN59196 | I+II+III | RPB8, POLR2H, RPABC3 | Polymerase Core | essential |
| EFN59231 | I+II+III | RPB12, POLR2K, RPABC4 | Polymerase Core | essential |
| EFN54945 | I+II+III | RPB5, POLR2E, RPABC1 | Polymerase Core | essential |
| EFN58319 | I+III | RPC19, POLR1D, RPAC2 | Polymerase Core | essential |
| EFN51752 | I+III | RPC40, POLR1C, RPAC1 | Polymerase Core | essential |
| EFN56557 | II | RPB3, POLR2C | Polymerase Core | essential |
| EFN56279 | II | RPB4, POLR2D | Initiation complex formation | dispensable |
| EFN55020 | II | RPB1, POLR2A | Active center | essential |
| EFN52187 | II | RPB2, POLR2B | Active center | essential |
| EFN57906 | III | RPC37, POLR3E | Initiation complex stabilization, start site selection | essential |
| EFN54463 | III | RPC11, POLR3K, RPC10 | RNA cleavage | essential |
| EFN52317 | III | RPC82, POLR3C, RPC3 | Open complex stabilization | essential |
| EFN52235 | III | RPC2, POLR3B | Active center | essential |
| *Downregulated genes* | | | | |
| EFN58415 | I | RPA12, ZNRD1 | RNA cleavage | dispensable |
| EFN53299 | I | RPA49, POLR1E | Initiation complex stabilization, start site selection | dispensable |
| EFN57002 | I+II+III | RPB6, POLR2F, RPABC2 | Polymerase Core | essential |
| EFN54224 | II | RPB7, POLR2G | Initiation complex formation | essential |
| EFN53258 | II | RPB9, POLR2I | RNA cleavage | dispensable |
| EFN56335 | III | RPC53, POLR3D, RPC4 | Initiation complex stabilization, start site selection | essential |
| EFN55967 | III | RPC25, POLR3H, RPC8 | Initiation complex formation | essential |

‡ as determined in Winzeler et al., 1999,
